# An unusual cause of upper gastrointestinal bleeding due to recurrent hepatocellular carcinoma: A case report

**DOI:** 10.1016/j.ijscr.2020.04.008

**Published:** 2020-05-11

**Authors:** Yuk Ho Liu, Eugene Yee Juen Lo, Kit Fai Lee, Charing Ching Ning Chong, Paul Bo San Lai

**Affiliations:** a Division of Hepatobiliary and Pancreatic Surgery, Department of Surgery, Prince of Wales Hospital, Hong Kong; b Department of Surgery, The Chinese University of Hong Kong, Hong Kong

**Keywords:** Hepatocellular carcinoma, Gastrointestinal bleeding

## Abstract

**Introduction:**

Upper gastrointestinal bleeding (UGIB) in patients suffering from hepatocellular carcinoma (HCC) is usually due to oesophageal or gastric varices secondary to portal hypertension. Very rarely, HCC can directly invade into the stomach or duodenum resulting in UGIB.

**Presentation of case:**

A 62-year-old man presented to the emergency department for haematemesis and tarry stool. He was a hepatitis B carrier and had received open radiofrequency ablation and wedge resection for HCC previously. Urgent endoscopy and contrast computed tomography confirmed recurrent HCC invading into the duodenum. The patient received radical resection and remained disease free for 7 years after the operation.

**Discussion:**

Direct invasion into gastrointestinal tract by HCC is rare. Different modalities of treatment have been reported in the literature with variable success. En-bloc resection should be considered if surgically feasible in order to achieve good haemostasis and possible long term survival.

**Conclusion:**

Upper gastrointestinal bleeding is a rare presentation of hepatocellular carcinoma and long term survival can be achieved by curative surgery.

## Highlights

• Hepatocellular carcinoma invading to gastrointestinal tract can present as bleeding.
• Invasion to gastrointestinal tract by hepatocellular carcinoma is relatively rare.
• Long term survival can be achieved by en bloc resection even if hepatocellular carcinoma invades into nearby organs.

## 1 Introduction

Patients with hepatocellular carcinoma (HCC) commonly have underlying chronic parenchymal liver disease or cirrhosis. Upper gastrointestinal bleeding (UGIB) in these patients are usually due to oesophageal or gastric varices secondary to portal hypertension. Very rarely, HCC can directly invade into the stomach or duodenum causing UGIB. Here we report a case of a recurrent HCC presented with haematemesis and tarry stool. The patient received radical resection of the tumour and remained disease free 7 years after the operation. The work has been reported in line with the SCARE criteria [1].

## 2 Presentation of case

**Fig. 1:**
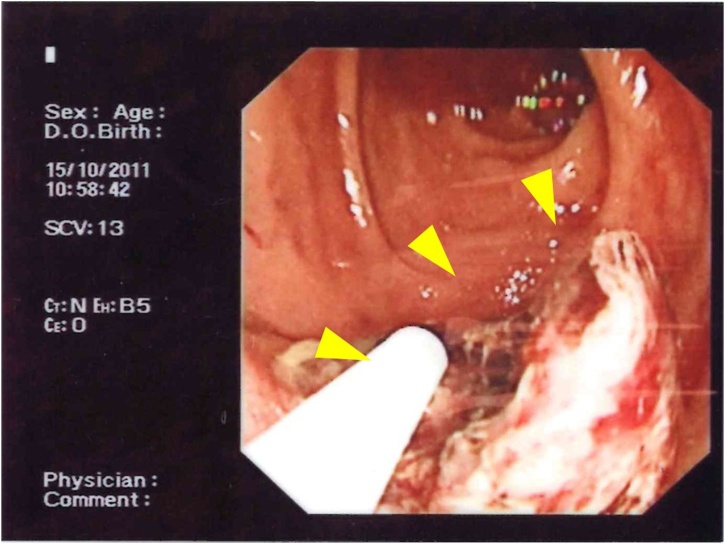
Endoscopic view of the duodenal ulcer (arrow).

**Fig. 2:**
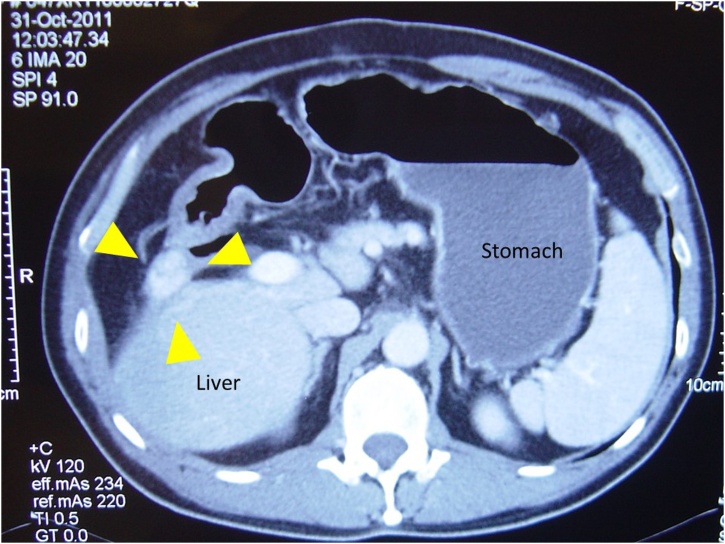
Computed tomography showing the tumour (arrow) invade to duodenum.

A 62-year-old man who was known to be a hepatitis B carrier and had a history of treated HCC presented as emergency with hematemesis and tarry stool. For the history of HCC, he has received open radiofrequency ablation and cholecystectomy for a segment V tumour seven years prior to the current presentation. Lamivudine was started post operatively after assessment by hepatologist and seroconversion of HBeAg was achieved one year later. Open wedge resection for two small recurrent tumours at segment VI was performed two years later. For the current admission, urgent upper endoscopy revealed a 1 cm irregular ulcer at posterior wall of first part of duodenum with adherent clot (Fig. 1). Adrenaline was injected to ulcer base and surrounding mucosa. Bleeding stopped after heat probe and hemoclip application. The patient was stabilised after endoscopic haemostasis. Urgent computed tomography (CT) was performed to exclude malignant infiltration of duodenum. It revealed a 2.4 cm recurrent hypervascular tumour at inferior part of right liver with local invasion to duodenum (Fig. 2). No other liver lesion was seen. The tumour marker alpha-fetoprotein (AFP) was all along not elevated.

**Fig. 3:**
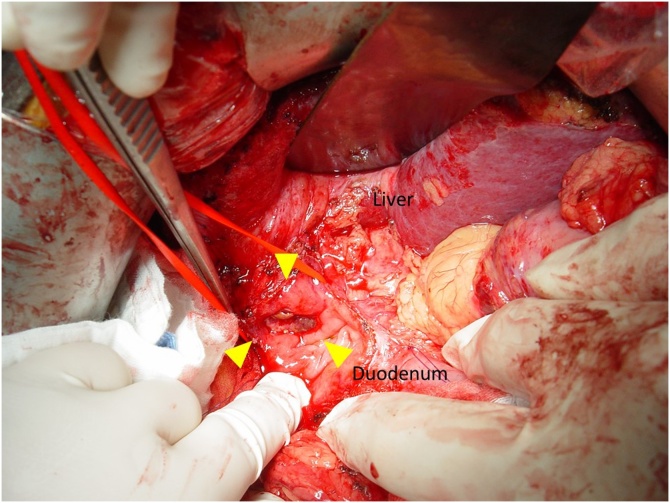
Operative view showing the tumour invading the posterior wall of duodenum forming an ulcer (arrow) after the duodenum was opened.

**Fig. 4:**
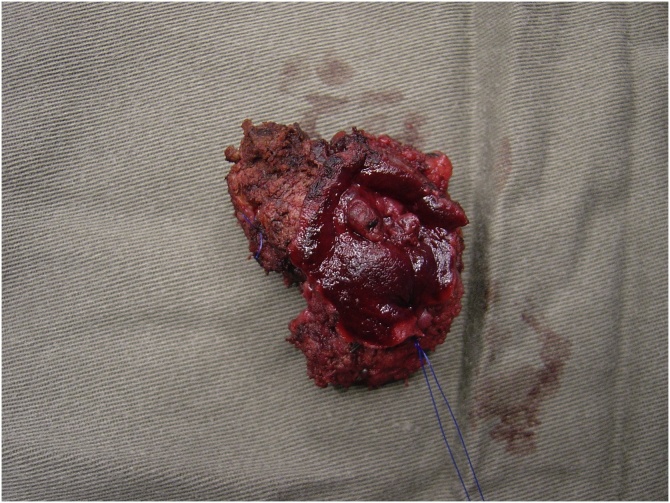
The resected specimen with cuff of duodenal wall.

Patient was offered laparotomy for resection of recurrent HCC. During operation, a 2 cm tumour was found arising from inferior part of segment V of liver directly invading into first and second part of duodenum. Wedge resection of the tumour together with a cuff of involved duodenum was performed (Fig. 3). The duodenal defect was closed primarily. Gastrojejunostomy was performed for the worry of duodenal stenosis at repair site. Operative blood loss was 800 mL and operation time was 7 h. Patient made an uneventful recovery. Pathology confirmed a 3.5 cm moderately differentiated HCC with invasion to duodenal wall associated with duodenal ulcer (Fig. 4). Patient was followed up regularly with disease surveillance by ultrasound and CT. Patient remained disease free and HBV DNA remained non detectable seven years after the last operation.

## 3 Discussion

HCC is well known to be a vascular tumour and has the tendency to invade to adjacent vascular structures like the portal vein, the hepatic vein or even the inferior vena cava [2]. Direct invasion of tumour to adjacent organs like gallbladder, diaphragm, stomach, duodenum and colon have been reported, especially for large size tumour [3]. Direct invasion to gastrointestinal tract was rare, and was reported to occur in 0.5–2 % of all clinical HCC cases [4]. The most common site of direct tumour invasion of the gastrointestinal tract was stomach followed by duodenum and colon [5]. Gastrointestinal bleeding and gastric outlet obstruction are rare presentations when duodenal invasion occurred [6]. Even more rarely, bleeding from HCC metastasis to gastric and jejunal wall have also been reported [7,8].

UGIB due to direct HCC invasion of the gastrointestinal tract can be difficult to manage as the involved mucosa have malignant infiltration. Usual endoscopic haemostatic measures can provide temporary effect but rebleeding is likely. Successful endoscopic treatment with ethanol injection has been reported [9]. People have also tried transarterial embolization of the supplying vessel to the involved tumour with success [5]. Lastly, successful haemostasis with radiotherapy for HCC invading to duodenum has also been reported [10].

The predisposing factors for gastrointestinal tract involvement were large liver lesions (>5 cm), subcapsular location and exophytic growth pattern [5]. In this particular case, the recurrent tumour was relatively small but still invaded to adjacent duodenum. This was likely due to previous surgery particularly open radiofrequency ablation which has caused inflammatory response and adhesion of adjacent bowel wall. Given the preserved liver function, small size and peripherally located tumour, it was suitable for local wedge resection together with the invaded duodenal wall. A long-term disease free survival was achieved with curative resection and long term antiviral therapy.

Successful resection combined with gastrectomy or duodenectomy have been reported in the literature [11,12]. Surgical resection not only stops the bleeding but also provides chance of cure for patients. Long – term survival was also reported after en-bloc resection for HCC invading stomach and duodenum [13]. Hence, local invasion to gastrointestinal tract should not constitute a contraindication for radical resection for HCC when combined resection of the involved gastrointestinal tract is feasible.

## 4 Conclusion

Upper gastrointestinal bleeding is a rare presentation of hepatocellular carcinoma and long term survival can be achieved by curative surgery.

Written informed consent was obtained from the patient for publication of this case report and accompanying images. A copy of the written consent is available for review by the Editor-in-Chief of this journal on request.

## Declaration of Competing Interest

All authors report no conflicts of interest.

## Sources of funding

All authors report no source of funding in this study.

## Ethical approval

The submitted study is not a research study.

## Consent

Written consent has been obtained from the patient.

## Author contribution

Study concept and design: Liu, Lee.

Data acquisition: Liu, Lo, Lee.

Data analysis and interpretation: N/A.

Drafting of the manuscript: Liu, Lee.

Critical revision of the manuscript for important intellectual content: Lo, Chong, Lai.

Statistical analysis: N/A.

Obtained funding: N/A.

Administrative, technical, or material support: N/A

Study supervision: Lee, Chong, Lai.

## Registration of research studies

The submitted study is not a research study.

## Guarantor

Liu.

## Provenance and peer review

Not commissioned, externally peer-reviewed.
